# Propagating acoustic waves on a culture substrate regulate the directional collective cell migration

**DOI:** 10.1038/s41378-021-00304-8

**Published:** 2021-11-11

**Authors:** Chikahiro Imashiro, Byungjun Kang, Yunam Lee, Youn-Hoo Hwang, Seonghun Im, Dae-Eun Kim, Kenjiro Takemura, Hyungsuk Lee

**Affiliations:** 1 Institute of Advanced Biomedical Engineering and Science, Tokyo Women’s Medical University, Shinjuku, Japan; 2 Department of Mechanical Engineering, Keio University, Yokohama, Japan; 3 School of Mechanical Engineering, Yonsei University, Seoul, Korea

**Keywords:** Electrical and electronic engineering, Microfluidics

## Abstract

Collective cell migration plays a critical role in physiological and pathological processes such as development, wound healing, and metastasis. Numerous studies have demonstrated how various types of chemical, mechanical, and electrical cues dictate the collective migratory behaviors of cells. Although an acoustic cue can be advantageous because of its noninvasiveness and biocompatibility, cell migration in response to acoustic stimulation remains poorly understood. In this study, we developed a device that is able to apply surface acoustic waves to a cell culture substrate and investigated the effect of propagating acoustic waves on collective cell migration. The migration distance estimated at various wave intensities revealed that unidirectional cell migration was enhanced at a critical wave intensity and that it was suppressed as the intensity was further increased. The increased migration might be attributable to cell orientation alignment along the direction of the propagating wave, as characterized by nucleus shape. Thicker actin bundles indicative of a high traction force were observed in cells subjected to propagating acoustic waves at the critical intensity. Our device and technique can be useful for regulating cellular functions associated with cell migration.

## Introduction

In living organisms, directional cell migration plays a key role in various physiological and pathological processes^1^. During cortical development in the embryonic stage, neurons migrate from the ventricular surface to the pial one in the cortex^2^. Disruption of neural cell migration is closely associated with critical diseases in the central nervous system^3^. In cancer metastasis, cells from the primary tumor tissue migrate into neighboring blood vessels, are transported through the blood, and penetrate into other tissues in the body^4^. Inhibition of phosphoinositide-dependent kinase-1 can reduce cancer cell motility and the invasion of tumor cells into neighboring tissues in vivo^5^. During wound healing, a group of cells migrate toward the wound site, proliferate, and cover the site^6^. It was reported that inhibition of vimentin reduced cellular motility without affecting proliferative capacity^7^, and wound healing was impaired in vimentin-deficient mice^8^. On the other hand, the wound healing rate was accelerated when cellular motility was increased by valproic acid treatment^9^. As demonstrated in numerous studies, the regulation of cell migration is essential for understanding the mechanisms of both physiological and pathological processes.

Cell migration is regulated by external cues determined by a chemical gradient, electrical field, or geometric pattern in the tissue microenvironment^6,10–16^. Fibroblasts were recruited to a wound site in response to the concentration gradient of platelet-derived growth factor-AA^6,10^. Electrical fields applied to fibroblasts induced the polarization of cells, thereby providing migration guidance^11^. Application of an electrical field increased the speed of wound repair by promoting cell migration toward wound sites^12^. In contrast to a nonpatterned substrate, cells cultured on a micropatterned substrate migrated along the direction of the micropatterns^13^. When the aligned scaffold was placed between the distal and proximal nerve stumps of a disconnected nerve, cells migrated along the aligned scaffold to regenerate the injured nerve^14^. However, the control of cell migration using chemical, electrical, and geometric stimuli has several limitations that have prevented its potential as a therapeutic method. For chemical-based regulation, a drug administered to a target tissue can diffuse into the surrounding tissues, leading to side effects, as observed in brain tissue subjected to treatment with anticancer drugs^15^. Due to the low conductivity of tissue, it is difficult to apply an electrical field to deep tissue under the skin^16^. Providing cells with geometric cues demands an incision to transplant an engineered scaffold^14^. Recent studies have revealed that mechanical stimulation can regulate cell migration by modulating the force balance in a cell^17^. A driving force for cell migration is primarily generated by the actomyosin system in the cytoskeleton^18^. When a flow was applied to a cell monolayer, the shear force altered the force balance between cells, which determined the direction and speed of migration^19^.

Acoustic waves are emerging as alternative cues to regulate cellular functions due to the following advantages^20,21^: (i) easy application to tissues in vivo, (ii) biocompatibility, (iii) noninvasiveness, (iv) directionality, and (v) fast delivery. Exposure to low-intensity pulsed ultrasound increased proliferation and subsequent differentiation of C2C12 cell into myotubes^22^. Pulsed ultrasound induced robust reversal behaviors in *Caenorhabditis elegans* via mechanosensitive ion channels activated by acoustic radiation force^23^. Recent studies have revealed that the speed of cell migration can be increased by the application of an acoustic wave via upregulated expression of migration-related proteins^24^. However, in a previous study on cell migration, it was difficult to modulate the migration direction, and the superposition of acoustic waves interrupted cell migration at nodes where the acoustic potential energy was locally minimized^24,25^.

Here, we studied how directional cell migration was governed by propagating acoustic waves. We developed a technique in which a propagating acoustic wave was applied to the direction of cell migration using a surface acoustic wave (SAW) device, which has been widely utilized to precisely manipulate microparticles, cells, and liquid samples^26–30^. Experiments using varied acoustic waves revealed that the propagating acoustic wave can enhance or suppress the speed of directional cell migration depending on the wave intensity. We found that altered cell migration was attributed to cytoskeletal reorganization with changes in actin stress fiber formation and nucleus orientation. The results of our study can be applied to regulate the migration of various types of cells using acoustic waves.

## Results

### Characterization of the acoustic wave device

**Fig. 1:**
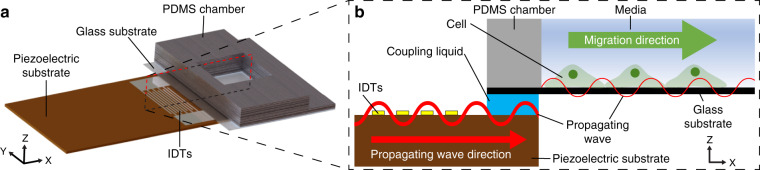
Schematic of the experimental setup. **a** 3D view of the device. **b** XZ cross-sectional view of the device

Our device is comprised of a piezoelectric substrate made of 128° Y-cut lithium niobate (LiNbO_3_), interdigital transducers (IDTs), and a polydimethylsiloxane (PDMS) chamber with a glass substrate (Fig. 1a). Glycerol solution, which functions as a coupling liquid, was injected between the piezoelectric substrate and the glass substrate (Fig. 1b). The thickness of the glycerol was regulated to minimize the attenuation of acoustic waves caused by a high acoustic attenuation coefficient^31^. In this study, the glycerol thickness was measured to be 40 μm using a laser scanning confocal microscope (Supplementary Fig. 1).

A SAW with a frequency of 14 MHz was generated by applying the radiofrequency (RF) signal to the IDTs in the device and was transferred to the coupling liquid in the form of a bulk acoustic wave. The transferred wave propagated along the glass substrate where cells were seeded.

**Fig. 2:**
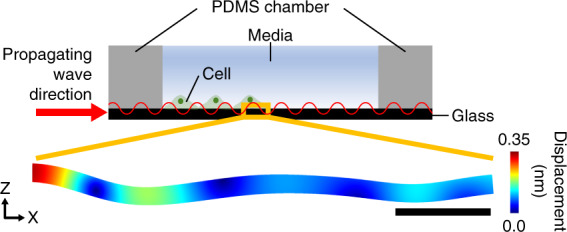
Computational simulation of the propagation of acoustic waves through glass. Schematic of the cell culture system with propagating acoustic waves and the estimated displacement of the glass substrate at the leading edge of the cell monolayer in the simulation. The applied voltage was 18 V. The scale bar represents 100 μm. The color bar represents displacement

We developed a two-dimensional finite element method (FEM) model of the SAW device using COMSOL Multiphysics 5.3a software. Briefly, the model consisted of a piezoelectric substrate, glycerol, glass, and a PDMS chamber with the same dimensions as the experiments and cell medium. An electric signal was applied to the IDTs placed on the piezoelectric substrate. The generated SAW was transferred to the glycerol layer, the glass substrate, and the cell medium, through the fluid–solid interface at which the pressure in the fluid domain was coupled with the stress in the solid domain (for details, see “Computational analysis of the SAW device” in the “Materials and methods” section). FEM simulation was used to analyze the displacement of the cell culture substrate of glass along which the transferred waves were propagated (Fig. 2, Supplementary Movie 1). The maximum displacement of the glass under the cell monolayer at the leading edge was estimated to be ~0.24 nm under our experimental condition of 18 V (Supplementary Fig. 2). The FEM simulation revealed the estimated sensitivity of the device, defined as the maximum displacement proportional to the applied voltage, to be 0.013 nm/V (Supplementary Fig. 2).

**Fig. 3:**
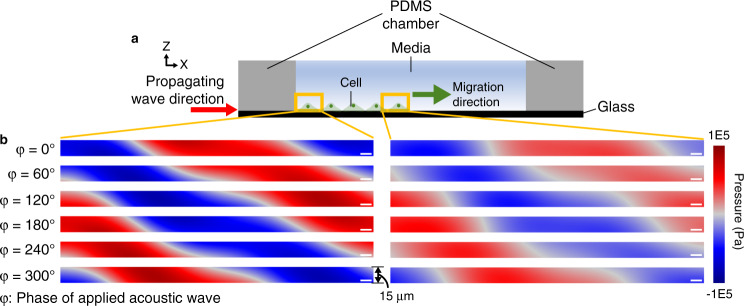
Propagation of acoustic pressure waves in the cell medium above the glass substrate. **a** Schematic of the cell culture system with propagating acoustic waves. **b** Propagation of the pressure wave in the culture medium near the wall of the PDMS chamber (left) and near the leading edge of the cell monolayer (right). φ represents the phase of the applied acoustic wave. Scale bars represent 10 µm. The height of the analysis window is 15 μm, similar to that of fibroblast cells adhered on the glass substrate^51^

The acoustic pressure distribution as a function of the wave phase revealed how the acoustic pressure field propagated from the wall of the PDMS chamber along the direction of cell migration in the culture medium, (Fig. 3, Supplementary Movie 2). The magnitude of the pressure field was higher in the region near the PDMS wall than in the region near the leading edge of cell (Fig. 3b), indicative of acoustic wave attenuation along the glass substrate. The pressure amplitude in the region above the leading edge of the cell monolayer, measured using a needle hydrophone, was similar to that estimated with the simulation at applied voltages of 2, 4, 6, 8, 10, 12, 14, 16, and 18 V (Supplementary Fig. 3). The average acoustic intensity applied to the cell monolayer was estimated to be 59.3 mW/cm^2^ at 18 V. The acoustic power was ~0.23 μW for a single cell with a presumed area of 400 μm^2^.

We also characterized the streaming flow in our device by tracking the motion of 1-μm-diameter polystyrene microparticles. We found that propagating waves produced circulating flow in the cell culture medium (Supplementary Fig. 4a). Therefore, the flow direction in the lower part of the medium was opposite to that in the upper part. We estimated that the speed of the counterflow at the surface of the cell monolayer to be ~56 µm/s at a maximum voltage of 18 V (Supplementary Fig. 4b).

### Effect of acoustic waves on the speed of cell migration

**Fig. 4:**
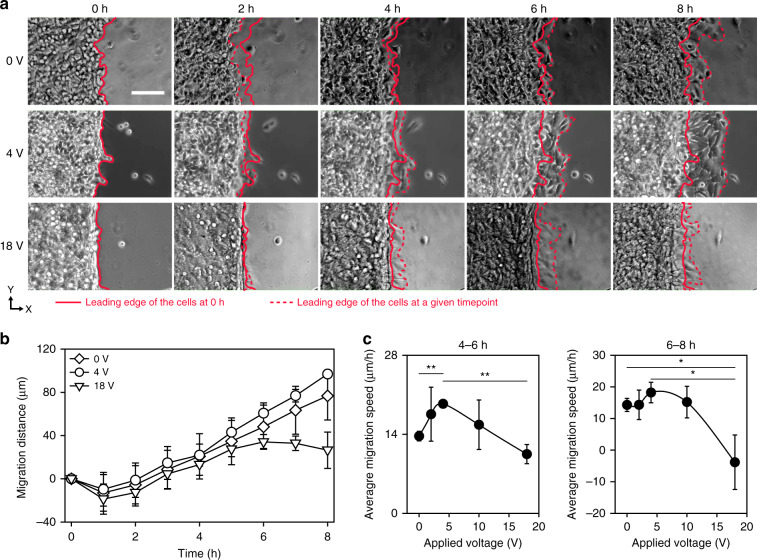
Effects of propagating acoustic waves on cell migration. **a** Time-lapse images of cell migration in response to acoustic wave stimulation at voltages of 0, 4, and 18 V. The scale bar represents 100 µm. The red line represents the leading edge of the cell monolayer at 0 h at each voltage. **b** Cell migration distance after removing the PDMS block at 0, 4, and 18 V (*n* = 3). **c** Migration speed at the 4–6 h and 6–8 h periods. The data are presented as the mean ± standard deviation (*p < 0.05 and **p < 0.005 versus each group, measured via one-way ANOVA, *n* = 3)

Using the developed experimental setup combining the SAW device and a cell culture chamber (Fig. 1), we investigated the effect of SAW on cell migration. Briefly, cells were seeded in one-half the region of the glass substrate on which an PDMS block was placed. After 8 h of culture to ensure the cells formed a monolayer, the cells were allowed to migrate to the intact region upon removal of the PDMS block (for details, see “Cell migration experiment” in the “Materials and methods” section). For the hour immediately after PDMS block removal, shrinkage of the cell monolayer was observed, as indicated by the retraction of the leading edge of the cell monolayer (Fig. 4a, Supplementary Movie 3). This retraction was attributed to the sudden alteration of the force balance upon removal of adhesion sites formed on the PDMS block. Cells adhered not only to the glass substrate but also to the PDMS block via the extracellular matrix. When the PDMS block was removed, the tension produced at the interface between the cells and the PDMS block disappeared, and the force balance was disrupted, leading to shrinkage of the cell monolayer. After 2 h, the leading edge of the cell monolayer moved forward in the direction of the propagating acoustic wave. In the absence of a SAW, the migration distance linearly increased with time (Fig. 4b). The average migration speed was estimated to be ~14 µm/h, which was similar to that measured in previous studies^32^. There was no significant difference in the migration distance between cultures subjected to 0, 4, and 18 V. However, after 4 h, the migration distance was significantly increased when the acoustic wave was applied at 4 V compared to when it was applied at 0 V. At 18 V, the migration distance was reduced for the entire period of the experiment compared to that induced with 0 V. In particular, a significant drawback of the cell monolayer was observed after 6 h, indicative of suppressed cell migration.

To investigate the effect of acoustic waves on cell migration as a function of acoustic wave magnitude, we compared the migration speed at when 0, 2, 4, 10, or 18 V was applied (Fig. 4c). At 4–6 h, compared to 0 V, the migration speed increased by 28 and 42% when the SAW was applied at 2 and 4 V, respectively. However, when the applied voltage was higher than 4 V, the migration speed was reduced. Similar to that in the 4–6 h period, the migration speed was increased by SAW application at 4 V in the 6–8 h period. We found that the migration speed was negative at 18 V, which was indicative of a significant retraction of the cell monolayer. This retraction might have been due to the suppression of cell migration caused by the high intensity of the acoustic waves. In addition, we observed the detachment of the cell monolayer from the glass substrate at the highest voltage applied in our experiments: 18 V (Supplementary Fig. 5, Supplementary Movie 4). Significant differences were found between 0 and 4 V and between 4 and 18 V at 4–6 h and between 0 and 18 V and between 4 and 18 V at 6–8 h (Fig. 4c).

### Effect of acoustic waves on the nucleus orientation and cytoskeletal structure

**Fig. 5:**
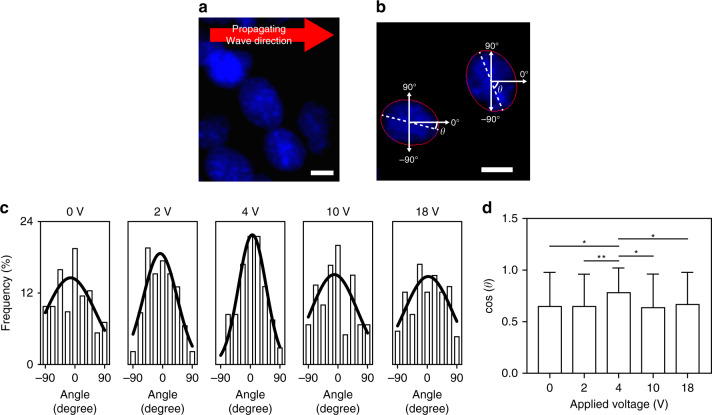
Effect of propagating acoustic waves on the orientation of cell nuclei. **a** Fluorescent images of cell nuclei at 0 V. The scale bar represents 10 µm. **b** Image processing for determining the nuclei orientation. The solid red line represents the ellipse fit to the nucleus. The orientation of the nuclei is defined as angle (*θ*) of the major axis of the nucleus relative to the direction of the propagating acoustic wave. The scale bar represents 10 µm. **c** Distribution of the nucleus orientation at various applied voltages of 0, 2, 4, 10, and 18 V (*n* = 46–113). The solid lines represent the Gaussian curves fitted to the angle distribution. **d** Cosine of the nucleus angle at various applied voltages (*p < 0.05 and **p < 0.005 versus each group via one-way ANOVA, *n* = 46–113). The cosine of the angle represents the degree of the nucleus alignment to the propagating wave direction

We investigated the effect of acoustic waves on the orientation of the cell nucleus, which is associated with various cellular functions, such as migration and differentiation^33^. The nucleus was imaged with a fluorescence microscope (Fig. 5a), and its orientation was characterized by defining the relative angle to the direction of the acoustic wave at 8 h of SAW application (Fig. 5b). Without acoustic wave application, the nuclei were aligned along the direction of cell migration to a certain degree, as shown in the distribution presented in Fig. 5c^33^. Compared to that at 0 V, the frequency at an angle of 0 increased in the orientation distribution at 2 and 4 V (Fig. 5c). This outcome was due to the increased population of nuclei aligned along the direction of wave propagation in response to SAW application. In contrast, at voltages higher than 10 V, the nucleus alignment was reduced, as indicated by the decreased frequency at angle 0, and the distribution was similar to that without SAW (Fig. 5c). To quantify the degree of the nucleus alignment, we calculated the cosine value of the nucleus angle, which approaches 1 when the nucleus is perfectly aligned along the wave direction (Fig. 5d). The average cosine value of the angle was 0.78 at 4 V, which was higher than that measured under other conditions. This result suggests that the nuclei are aligned primarily along the direction of propagating wave at 4 V.

The nucleus alignment as a function of applied voltage was consistent with the changes in migration distance, thereby suggesting that the applied acoustic wave dictated collective cell migration by regulating the orientation of the nuclei.

**Fig. 6:**
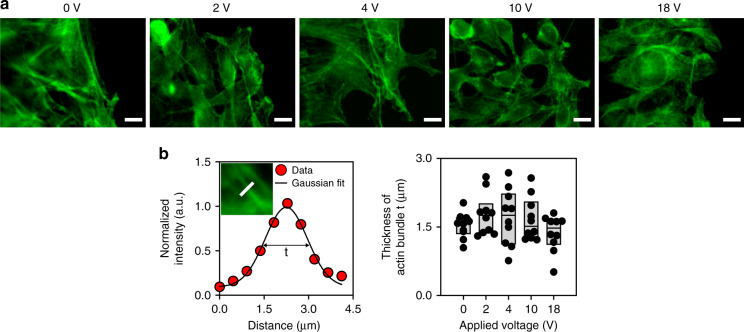
Effect of propagating acoustic waves on the actin cytoskeleton. **a** Fluorescent images of actin at various applied voltages. The scale bars represent 10 µm. **b** (Left) Normalized intensity of F-actin in a cell along the white line in the inset image and (right) thickness of actin bundle *t* at various applied voltages (*n* = 10). The thickness of bundle *t* was defined as the full width at the half maximum of Gaussian curve fitted to the normalized intensity. In the plot showing the thickness of the actin bundle, the upper, middle, and bottom lines of the boxes represent the 25th, 50th, and 75th percentiles of the data

We investigated the effect of acoustic waves on the cytoskeletal structure of actin. As shown in the fluorescent images, actin stress fibers were found in cells subjected to acoustic waves when 0, 2, or 4 V was applied. In contrast, actin filaments appeared to be dispersed, and stress fibers were rarely observed in cells subjected to acoustic waves at high voltages, that is, 10 and 18 V (Fig. 6a). The thickness of actin bundles in an image was estimated by measuring the width of the Gaussian curve of the fluorescence intensity fitted to the normalized intensity (Fig. 6b). Compared to that at 0 V, the thickness of actin bundles appeared to be increased at 2 and 4 V. When the applied voltage was higher than 10 V, the bundle thickness was significantly reduced (Fig. 6b). We found that the variation of actin bundle thickness as a function of the applied voltage was consistent with the change in migration speed, which is shown in Fig. 4, indicating that the reduced migration at a high voltage might be attributable to the altered cytoskeleton structure with diminished actin bundle formation.

## Discussion

We investigated the effect of acoustic waves on cell migration. Compared to a previous study where cells were cultured directly on top of a piezoelectric substrate^34^, our technique was advantageous for the quantitative analysis of cell migration, as the direction of cell migration was parallel to the direction of the acoustic wave. In addition, in our experimental setup, the effect of the electrical field on cell migration was negligible as the cells were cultured on nonconductive material. When acoustic waves propagate in cell medium via the glass substrate, heat and streaming flow can be generated, which may affect cell migration. To prevent damage to cells by increasing heat, the temperature was regulated to be maintained below 36.5 °C during acoustic wave application using a feedback loop control (Supplementary Fig. 6). The corresponding shear stress produced by the streaming flow (Supplementary Fig. 4) on the cell membrane was estimated to be ~3.7 mPa, which was much smaller than the critical stress of ~2000 mPa required for altering the migration speed of confluent fibroblasts^35^. We presumed that the counterflow on the cells did not have a significant effect on cell migration as the produced shear stress would be relatively low due to the low speed. Taken together, the data show that the change in migration speed observed in our study was primarily caused by the propagating acoustic wave.

The effect of acoustic wave application on cell migration can differ by cell type, assay condition, and wave characteristics. In previous studies, SAW application increased the surface area migration rate for MDCK-II cells by 135%^36^, while it increased the rate for SaOs-2 cells by only 15%^34,36^. In our study, the migration rate of 3T3 fibroblasts was increased by 42% upon SAW application. The migratory behavior of cell can also depend on the type of assay performed. Stamp et al. and Brugger et al. performed a wound healing assay in which cells migrated from two edges to close the gap^34,36^. The cell migration was bidirectional, and paracrine signaling between two separated regions of cells played a role in determining the migration speed in these wound healing assays. In contrast, in our assay, cells migrated into a cell-free area. In addition, it had been previously demonstrated that the migration behavior of the cells can be modulated by the amplitude and wavelength of the SAW^36^.

In our study, a significant change in migration speed occurred 4–6 and 6–8 h after SAW application, as shown in Fig. 4. Experiments to investigate the effect of acoustic waves on cell migration were recently conducted using wound healing assays, and the results showed that the gap between cells was covered after 24 h^34,36^. Even in these previous studies, a significant increase in the cell migration speed was observed in the first few hours after the SAW was applied. Cells can respond to mechanical or acoustic stimulation faster than to biochemical stimulation. For example, the migration of mesenchymal stem cells was altered by shear stress applied for 1 h^37^. The migratory behaviors of these mesenchymal stem cells were altered by changes to the mechanical properties of the culture substrate within 10 h^38^. It seems that cell migration can be altered by acoustic cues faster than biochemical cues via mechanotransduction.

We analyzed the structure of the actin cytoskeleton and the orientation of the nuclei to elucidate the potential mechanism of cell migration regulated by acoustic waves. We found that the nucleus orientation was more frequently aligned along the direction of the propagating acoustic wave and that thicker actin bundles were observed in cells subjected to acoustic waves with intermediate intensity. The nucleus orientation can be determined by the mechanical forces generated in actin–myosin or microtubule–dynein interactions through the Rho signaling pathway^33^. The shape and location of the nucleus dictate the positioning of the microtubule-organizing center^33^ and the tensional balance within a cell^39^. Actin filaments play important roles in mediating intra- or intercellular forces in cells^18^. We observed thicker actin filament bundles in cells subjected to acoustic waves with critical intensity (Fig. 6). This result was consistent with the previous studies that demonstrated that low-intensity acoustic waves promoted actin bundle formation and focal adhesion protein localization in fibroblasts by activating Rho kinases^40^. In addition, propagating acoustic waves generate acoustic radiation forces on cells, which strengthens focal adhesions^41,42^. The promoted actin bundle and focal adhesion formation provides pivotal points for traction force, which is required for cell migration. On the other hand, when the applied voltage was higher than the critical value, the acoustic waves suppressed actin bundle formation in our study (Fig. 6). This outcome might have been a result of actin disruption, which can be caused by acoustic waves with high amplitude^43^. In addition, we found that the propagation direction of the acoustic waves had a component normal to the culture surface at a 22° angle (Fig. 3, Supplementary Movie 2). The formation of focal adhesions can be interfered by the lifting force^44^. When the intensity of the applied acoustic wave was high, we observed delamination of the cell monolayer (Supplementary Fig. 5), similar to that observed when the propagating acoustic wave was applied in a direction normal to the culture substrate^45^. We found delamination of the cell monolayer near the PDMS wall, where the acoustic pressure magnitude in the chamber was relatively high, as analyzed with the simulation (Fig. 3). This observation suggests that propagating acoustic waves with a high intensity suppress cell migration by disrupting the formation of cytoskeletal filaments and focal adhesion sites.

We demonstrated that the migration of cells cultured on a 2D planar substrate was regulated by acoustic waves propagating along the substrate. Our technique can be further applied to regulate cellular functions under various physiological or pathological conditions. The effect of acoustic waves on the migration of cancer cells in various tumor environments can be investigated to study metastasis. Since the response of cells to acoustic waves can depend on wave characteristics, further study using various wavelengths should be performed to better modulate cell migration. The characteristics of migrating cells are closely associated with their phenotype, which is critical for differentiation and maturation^46^. The regulation of cell migration using acoustic waves can be advantageous in developing a tool to fabricate biomimetic tissues by engineering cell functions.

## Conclusion

We developed a device consisting of a SAW and a fluidic chamber to investigate the effect of acoustic waves on the directional migration of cell monolayers. We demonstrated that the speed of cell migration increased when acoustic waves with critical intensity were applied. However, when the intensity of the applied acoustic wave was higher than the critical value, cell migration was suppressed. We showed that the changes in cell migration in response to the applied acoustic wave were correlated with the alteration of the cytoskeletal structure. Cells subjected to the propagating wave with the critical intensity exhibited the nucleus alignment and thick actin bundle formation. Our experimental results and acoustic technique can be used not only to understand cell migration itself but also to develop a technique for regulating cellular functions associated with cell migration.

## Materials and methods

### Preparation of the SAW device

A cell culture chamber was prepared by pouring PDMS solution (Sylgard 184, Dow Corning, USA) at a 10:1 mixing ratio of base agent:curing agent into a mold fabricated using a 3D printer (Objet30; Stratasys, Israel). After baking at 60 °C for 24 h, a cover glass was attached to the PDMS chamber by O_2_ plasma treatment (CUTE-MP, Femto Science, Republic of Korea).

The SAW device was composed of a 128° Y-cut LiNbO_3_ substrate and IDTs. Two sets of 23 IDT fingers were fabricated on the LiNbO_3_ substrate using a conventional photolithographic technique^26,27^. The fingers were designed to produce a SAW with a wavelength of 280 µm. The center frequency of the SAW was 14 MHz. To generate a propagating SAW without standing waves, foam tape (3702, 3M, USA) was placed near the edge of the LiNbO_3_ substrate to absorb waves and prevent their reflection. The SAW device was mounted on a microscope in a live cell imaging system (Live Cell Instrument, Republic of Korea). A cell culture chamber was added to the SAW device (Fig. 1a). Glycerol (G5516, Sigma-Aldrich, USA) as a coupling liquid was inserted between the SAW device and the PDMS cell culture chamber. The 3D height profile of the SAW device was obtained using a laser scanning confocal microscope (VK-X, Keyence, Japan). From the height profiles, the thickness of the glycerol layer was calculated to be 40 μm (Supplementary Fig. 1).

An RF signal was generated from a function generator (33622A, Keysight Technologies, USA), passed through an amplifier (LZY-22+, Mini-Circuits, USA), and applied to the IDTs. The voltages of the RF signals used in our experiments were 0, 2, 4, 10, and 18 V. The SAW was transferred via the coupling liquid and propagated through the glass substrate in the form of a Lamb wave^47^ (Fig. 1b). Thus, the cells seeded on the glass substrate were subjected to the propagating acoustic wave.

### Computational analysis of the SAW device

A two-dimensional model consisting of the SAW substrate, glycerol, glass, the PDMS chamber, and cell medium was developed using the FEM analysis software COMSOL Multiphysics 5.3a (COMSOL, Sweden) (Supplementary Fig. 7)^27,48,49^. The dimensions in the computational model were equal to those in the experimental device. The maximum size of the mesh of the model was **λ**/15, where *λ* is the wavelength in each domain. Parameters for the SAW device and glass domains were adopted from the material library in COMSOL Multiphysics 5.3a. Other parameters utilized in the simulation are listed in Supplementary Table 1.

The LiNbO_3_ domain was modeled as piezoelectric material using the “Electrostatics,” “Solid Mechanics,” and “Piezoelectric Effects” modules^49^. Cell medium, glycerol, and PDMS were modeled as a linear compressible fluid with acoustic attenuation using the “Pressure Acoustics” module^49^. Wave propagation was governed by the Helmholtz equation^48,49^.

In terms of electrostatics, domains except for the IDT fingers had electrical insulation boundary conditions^49^. A set of 23 IDT fingers had an electrical potential boundary condition, and the other set of IDT fingers had a ground boundary condition^27,49^. For solid mechanics and pressure acoustics, stress-free and pressure release conditions were applied to the boundaries of the solid and fluid domains, respectively^48,49^. The bottom and side boundaries of the LiNbO_3_ domain were assumed to be in a low reflection condition due to the absorption of the bulk acoustic wave by the conductive epoxy placed under the LiNbO_3_ substrate^50^. At the fluid–solid interfaces, the pressure in the fluid domain was assumed to be equal to the stress in the solid domain^27,49^.

Based on the FEM results, we estimated the acoustic intensity and power applied to the cells. We assumed that the pressure amplitude calculated on the basis of the two-dimensional simulation was constant along the direction normal to the plane of the model. The acoustic power applied to the cell monolayer was calculated by multiplying the acoustic intensity by the cell area.

### Characterization of the pressure amplitude in the cell culture chamber

We measured the pressure amplitude at the leading edge of the cell monolayer using a 1-mm-diameter needle hydrophone (Precision Acoustics, UK) connected to a preamplifier (Precision Acoustics, UK), DC coupler (Precision Acoustics, UK), and oscilloscope (DSO5014A, Agilent, USA) (Supplementary Fig. 3).

### Characterization of the flow field in the cell culture chamber

Taking into account the height of the fibroblasts used in our experiments^51^, the flow speed was evaluated to be 15 µm above the glass surface. Movement of fluorescent microparticles (F8823; Molecular Probes, Eugene, OR, USA; 1 µm in diameter) was captured with an epifluorescence microscope (Ni-U, Nikon, Tokyo, Japan) with a charge-coupled device (CCD) camera (DS-Qi1MC, Nikon). The flow field was evaluated by analyzing the trajectories of the fluorescent microparticles (F8823; Molecular Probes, Eugene, OR, USA) using the open-source software PIVlab V1.41^52^ in MATLAB R2017b software (MathWorks, Natick, MA, USA).

### Cell culture

NIH-3T3 fibroblasts (ATCC, Rockville, MD, USA) were cultured in growth medium (DMEM) (LM 011–05, Welgene, Republic of Korea) supplemented with 10% fetal bovine serum (16000–044, Gibco, USA) and 1% penicillin/streptomycin (15140-122, Gibco, USA) in an incubator at 37 °C and 5% CO_2_.

### Cell migration experiment

**Fig. 7:**
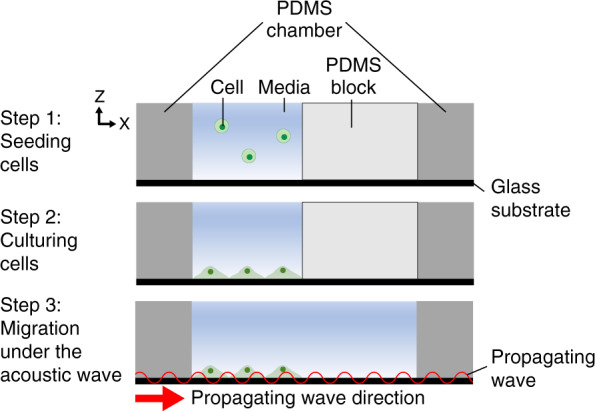
Experimental procedures. A cell solution was poured to fill one-half of a PDMS chamber (step 1). Cells were seeded to adhere to the glass substrate (step 2). The PDMS block was removed to allow the cells to migrate into the intact region (step 3). The arrow in step 3 indicates the direction of the propagating acoustic wave

The effect of the propagating acoustic wave on directional cell migration was investigated by modifying the wound healing assay protocol^32^. For sterilization, a PDMS cell culture chamber was immersed in 70% ethanol and exposed to UV light. A block made of PDMS was placed on the top surface of the glass substrate to ensure that one-half of the substrate remained intact during cell seeding (Fig. 7). A total of 4.0 × 10^5^ cells in 200 µL media were seeded onto the half of the glass substrate without the PDMS block. After 8 h of culture, the PDMS block was removed to allow cells to migrate toward the intact region. The acoustic wave was applied immediately after the removal of the PDMS block. Phase contrast images of the leading edge of the cell monolayer were taken every 5 min for 8 h. For capturing these images, an upright microscope (Ni-U, Nikon, Japan) with a CCD camera (DS-Qi1MC, Nikon, Japan) was used. To prevent damage to cells due to the heat generated by acoustic wave propagation^27^, the temperature was controlled using a live cell imaging system to remain in the physiological range of 36‒38 °C (Supplementary Fig. 6). The leading edge of the migrating cell monolayer was detected manually in an image using Fiji Software (National Institutes of Health, USA)^53^. The migration distance at a given time point was calculated as the difference between locations at two time points. The cytotoxicity of the acoustic wave was evaluated using a Live/Dead™ viability/cytotoxicity kit (L3224, Invitrogen, USA). The applied acoustic wave did not reduce cell viability under our experimental conditions (Supplementary Fig. 8).

### Immunostaining

Cells were fixed with 4% paraformaldehyde (15710, Electron Microscopy Sciences, Hatfield, PA, USA) and permeabilized with 0.5% Triton X-100 (X100, Sigma-Aldrich, USA) for 8 min. After washing with PBS, the cells were incubated with Hoechst 33342 (B2261, Sigma-Aldrich, USA) and Alexa-488 phalloidin (A12379, Invitrogen, USA) diluted in 1% BSA (B4287, Sigma-Aldrich, USA) blocking solution at a ratio of 1:200 for 1 h to stain the nuclei and actin filaments, respectively. The cells were mounted using ProLong^®^ Diamond Antifade Mountant (P36965, Invitrogen, USA), covered by a 9 mm square cover glass (72190-09, Bellco Glass, USA), and imaged using an epifluorescence microscope (Ni-U, Nikon, Japan) equipped with a CCD camera.

### Image analysis

The thickness of the actin bundle was estimated by analyzing the intensity profile in a fluorescent image using Fiji software (National Institutes of Health, USA)^53^. The fluorescence intensity was normalized by the maximum value and fitted to the Gaussian distribution curve. The thickness of actin bundles, *t*, was defined as the full width at half of the maximum normalized intensity^54^.

For the analysis of the nucleus orientation, a fluorescent image of nuclei was binarized and segmented manually using Fiji software (National Institutes of Health, USA)^53^. The shape of each nucleus was assumed to be an ellipse in the “Analyze Particles” function of the Fiji software^53^ (Fig. 5b). The orientation of a nucleus was defined as the angle of the major axis of the ellipse relative to the direction of SAW propagation. To quantify the extent of the nucleus alignment along the direction of the propagating wave, we calculated the cosine of the nucleus angle. The cosine varied in a range from 0 to 1, where 1 indicates that the nuclei are perfectly aligned.

### Statistical analysis

The data are presented as the mean ± standard deviation. The statistical significance was determined using one-way ANOVA.

## Supplementary information

Supplementary information

Supplementary Movie 1

Supplementary Movie 2

Supplementary Movie 3

Supplementary Movie 4
