# Supplementary material for: Propagating acoustic waves on a culture substrate regulate the directional collective cell migration
Source: Microsyst Nanoeng. 2021 Nov 11;7:90. doi: 10.1038/s41378-021-00304-8 (PMC8581020; doi:10.1038/s41378-021-00304-8)
Supplement: Supplementary file 1 — Supplementary information [file 41378_2021_304_MOESM1_ESM.docx]

**Supplementary Materials**

**Propagating acoustic waves on a culture substrate regulate**

**the directional collective cell migration**

*Chikahiro Imashiro^1,2*^, Byungjun Kang^3*^,* *Yunam Lee^3^,* *Youn-Hoo Hwang^3^,* *Seonghun Im^3^, Dae-Eun Kim^3^,* *Kenjiro Takemura^2^, and Hyungsuk Lee^3^*

*^1^Institute of Advanced Biomedical Engineering and Science, TWIns, Tokyo Women’s Medical University, 8-1 Kawada-cho, Shinjuku 162-8666, Japan*

*^2^Department of Mechanical Engineering, Keio University, 3-14-1 Hiyoshi, Kohoku-ku, Yokohama 223-8522, Japan*

*^3^School of Mechanical Engineering, Yonsei University, Yonsei-ro 50, Seodaemun-gu, Seoul 03722, Republic of Korea*

*^*^These authors contributed equally to this work*

Correspondence should be addressed to Hyungsuk Lee (E-mail: hyungsuk@yonsei.ac.kr, phone: 82-2-2123-5824, fax: 82-2-2123-8641)

E-mail address of authors

Chikahiro Imashiro: imashiro@keio.jp

Byungjun Kang: crbox@yonsei.ac.kr

Yunam Lee: yunamlee@yonsei.ac.kr

Youn-Hoo Hwang: yhhwang@yonsei.ac.kr

Seonghun Im: seonghun.im@yonsei.ac.kr

Dae-Eun Kim: kimde@yonsei.ac.kr

Kenjiro Takemura: takemura@mech.keio.ac.jp

Hyungsuk Lee: hyungsuk@yonsei.ac.kr

**Supplementary Table I. Parameters utilized in the computational simulation of the acoustic wave propagation**

| **Parameter** | **Value** |
| --- | --- |
| **Density of cell media** | **998 kg/m^3^** |
| **Sound of speed of cell media** | **1500 m/s** |
| **Attenuation coefficient of cell media** | **325.08 dB/m** |
| **Density of glycerol** | **1260 kg/m^3^** |
| **Sound of speed of glycerol** | **1930 m/s** |
| **Attenuation coefficient of glycerol** | **5378 dB/m** |
| **Frequency** | **14 MHz** |


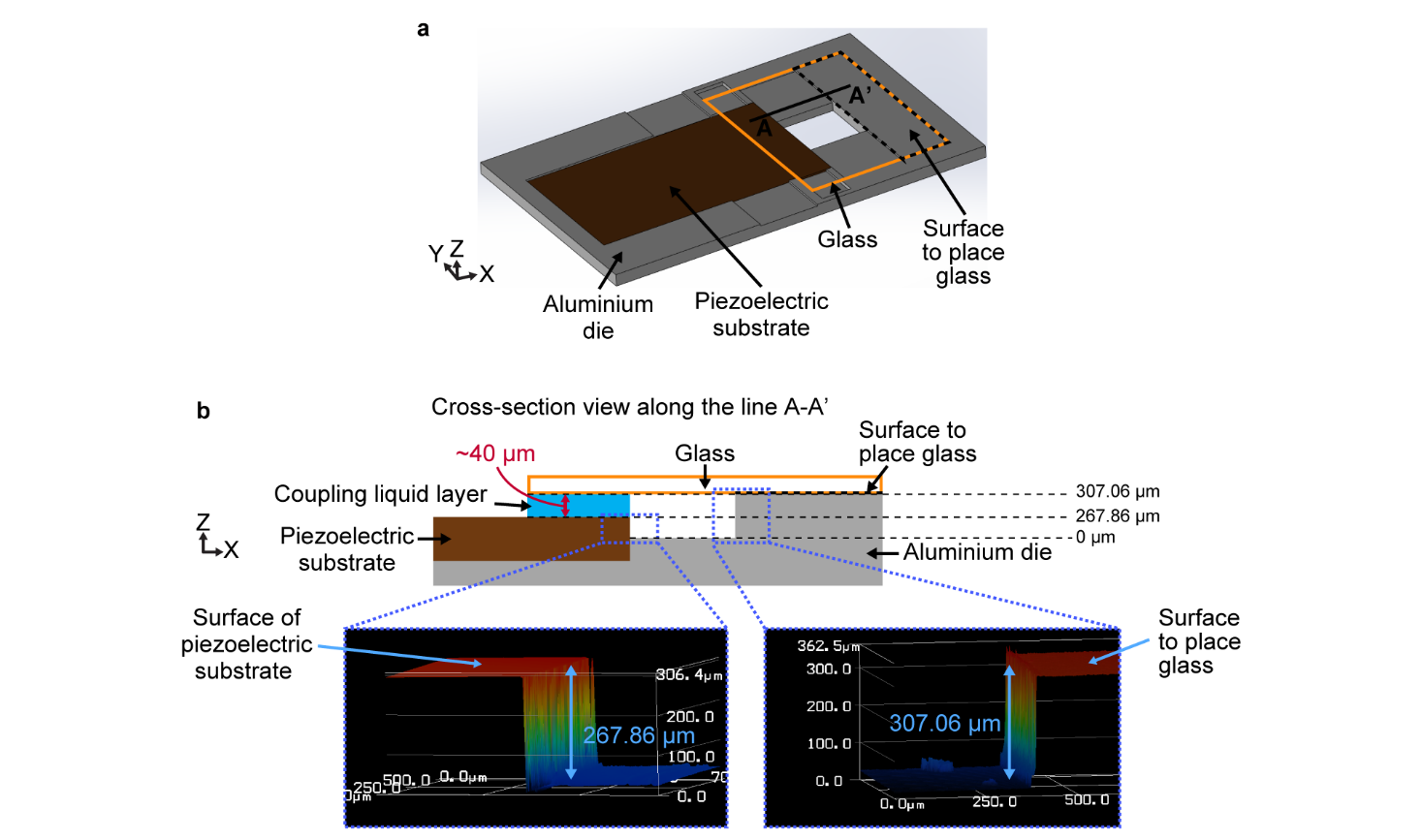


**Supplementary Figure 1. Thickness measurement of the glycerol layer.** a: 3D schematic of the SAW device comprised of aluminium die, piezoelectric substrate, and glass. b: (Top) Cross-section view of the device showing a coupling liquid layer between the piezoelectric substrate and glass. (Bottom) 3D height profiles to estimate the thickness of the coupling layer in the device.


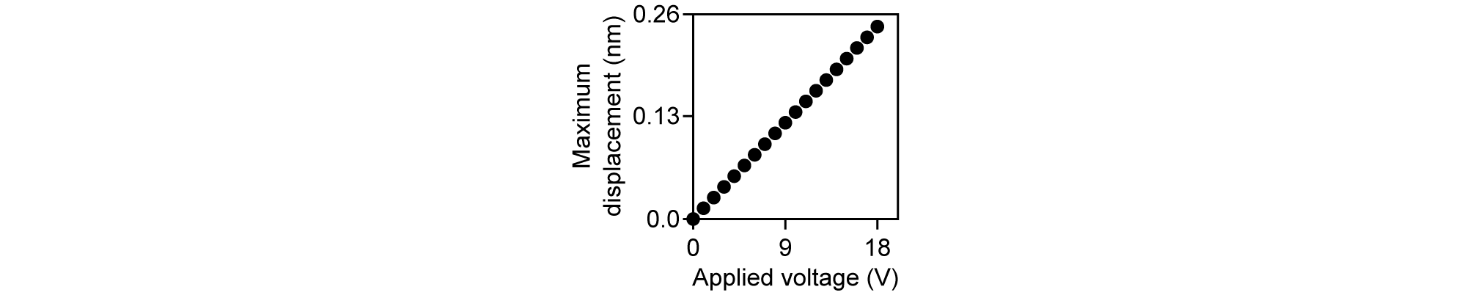


**Supplementary Figure 2. Maximum displacements of the glass at the leading edge as a function of applied voltage.**


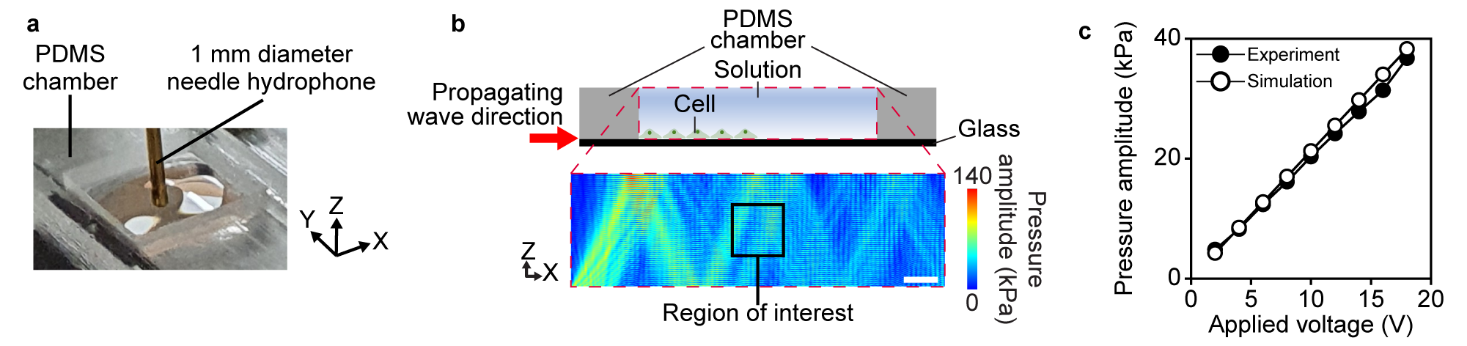


**Supplementary Figure 3. Comparison of the pressure amplitudes measured by a needle hydrophone and calculated from the FEM simulation.** a: The experimental setup showing a 1.0 mm diameter needle hydrophone in a PDMS chamber. b: A pressure amplitude distribution estimated from the FEM simulation. The rectangle in black lines shows the region of interest of 1.5 mm x 1.5 mm to calculate an average of pressure amplitude. Scale bar represents 1 mm. c: Comparison of pressure amplitude measured experimentally with that estimated numerically at varied applied voltages.


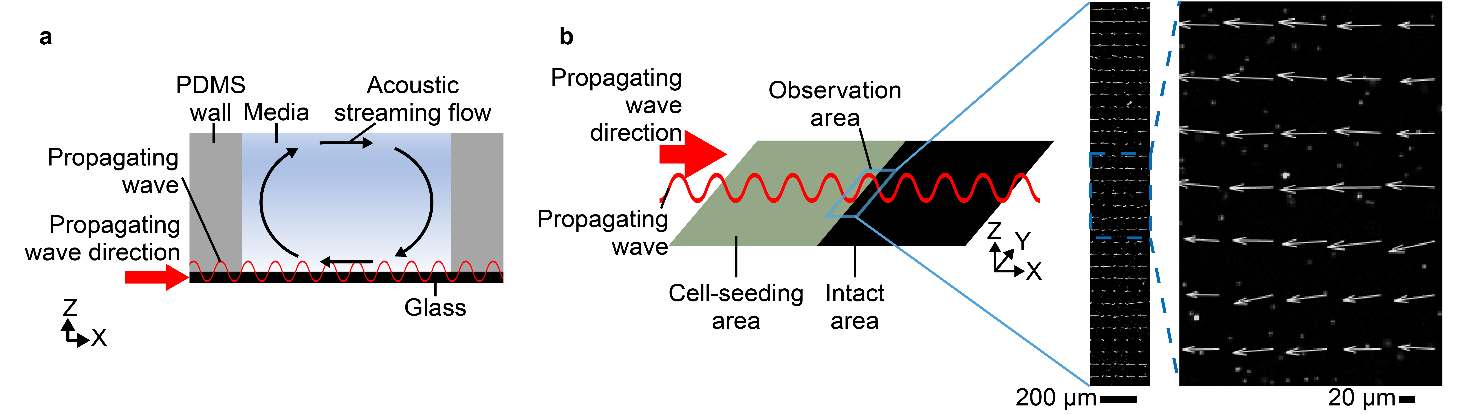
 **Supplementary Figure 4. Quantitative analysis for the flow produced by the propagating acoustic wave.** a: The side view shows the pattern of the acoustic streaming flow in the cell culture chamber. b: A flow velocity profile in the field of view at 18 V.


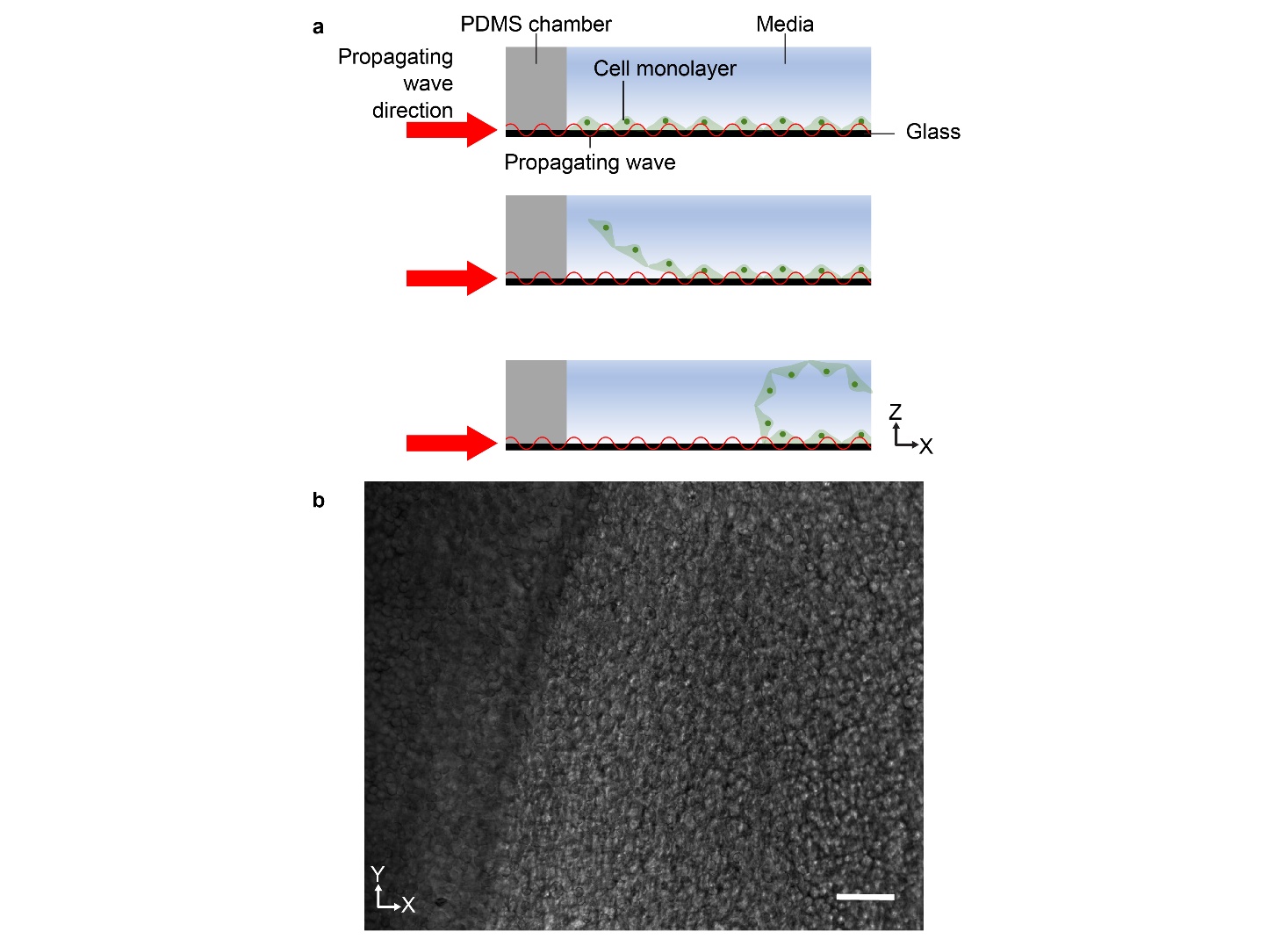


**Supplementary Figure 5. Delamination of the cell monolayer at 18 V.** a: Schematic of cell monolayer delamination. b: Phase contrast image of the delaminated cell monolayer treated with trypan blue. No cells were observed to be stained, suggesting that the detached cells were live. The scale bar represents 100 µm.


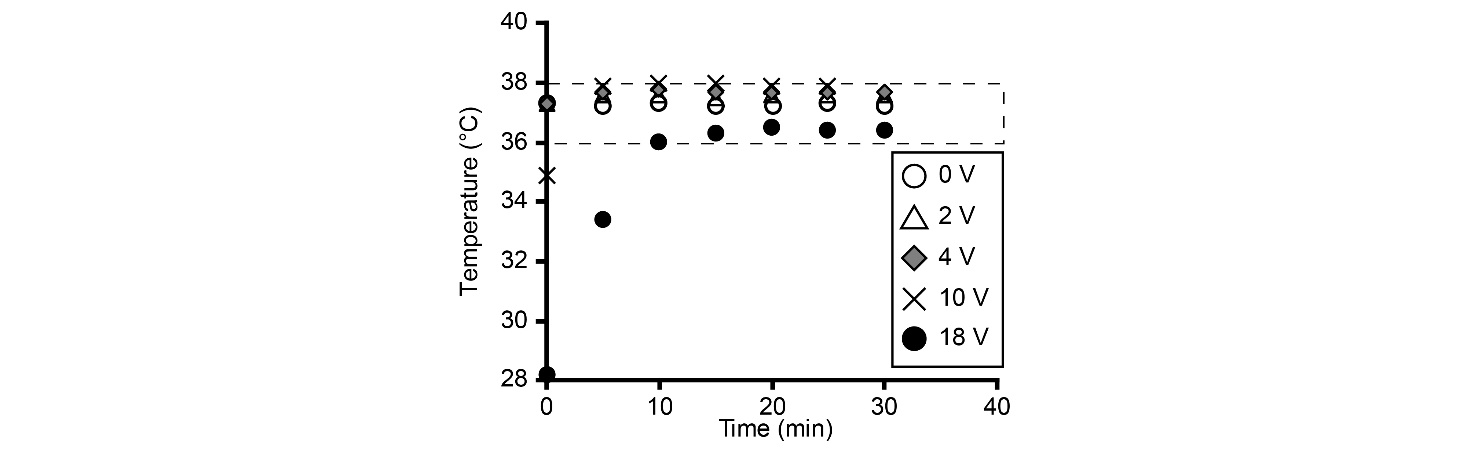


**Supplementary Figure 6. Time-dependent temperature of the cell media in the culture chamber at varied applied voltages.** Temperature was maintained at the physiological range of 36-38 ℃ within 10 min after control.


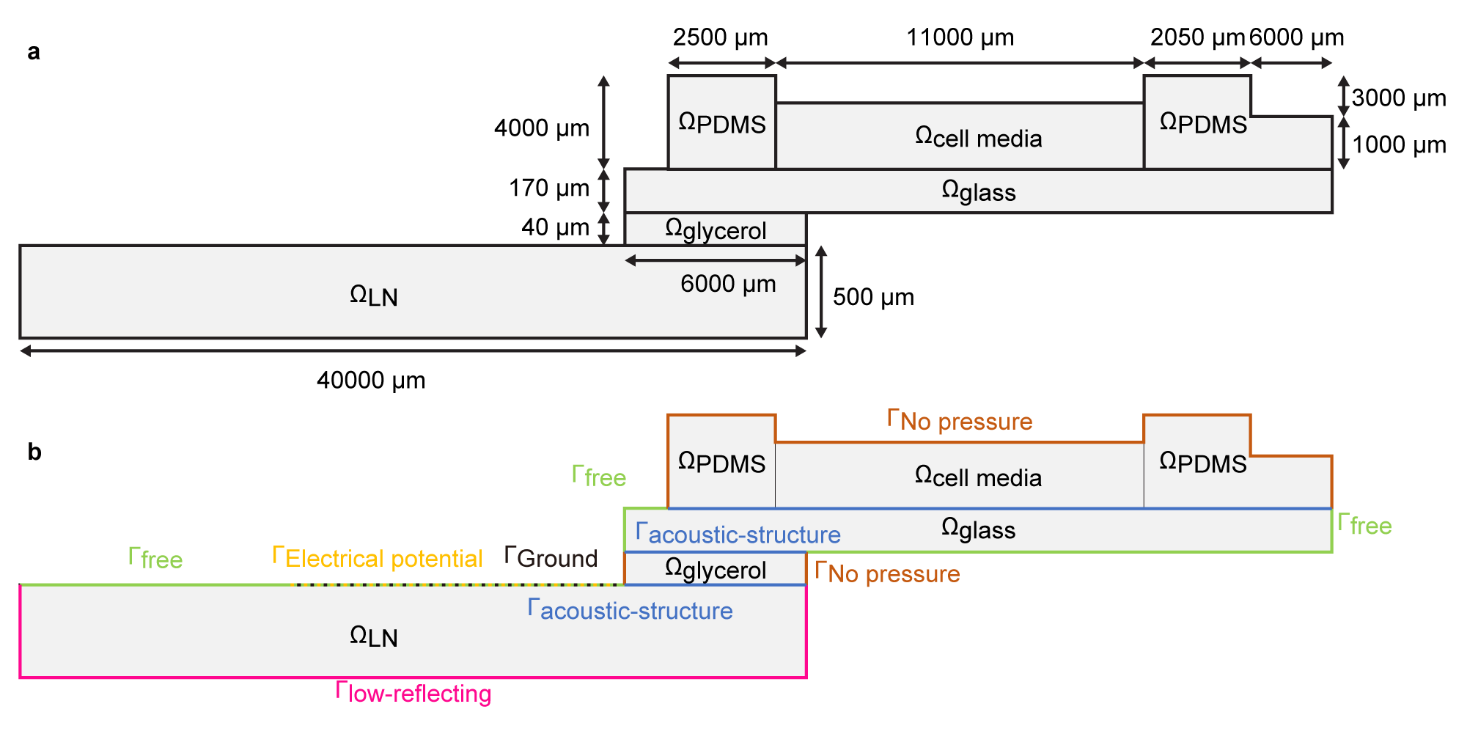


**Supplementary Figure 7. Two-dimensional computational model of the acoustic device combined with the PDMS cell culture chamber.** Schematics showing the dimensions (a) and boundary conditions (b) of each domain of the computational model (not to scale).


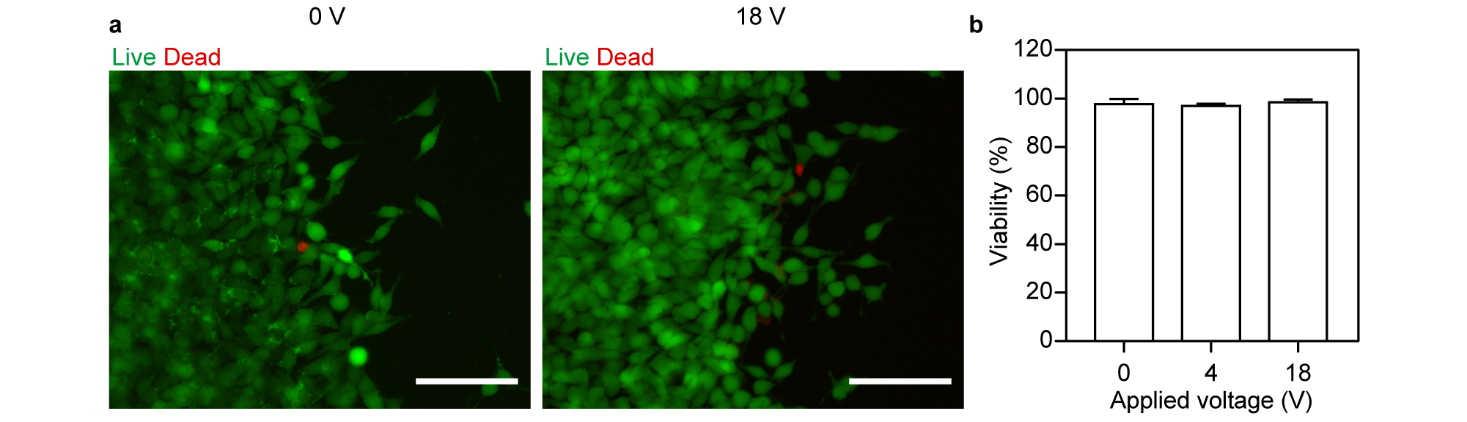


**Supplementary Figure 8**. Results of cytotoxicity assay. a: Live/dead images of cells with and without application of the acoustic wave at 18 V for 8 h. Scale bars represent 100 μm. b: Viability of the cells at varied applied voltages (*n* = 4).

**Legends of Supplementary Movies**

**Supplementary Movie 1. Displacement field in the glass substrate at the leading edge of the cell monolayer.**

**Supplementary Movie 2. Pressure field in the cell culture media near the PDMS chamber wall (top) and at the leading edge of cell monolayer (bottom).**

**Supplementary Movie 3. Migration of 3T3 fibroblasts in response to acoustic wave stimulation at voltages of 0, 4, and 18 V.**

**Supplementary Movie 4. Delamination of the cell monolayer at 18 V in the period of 6-8 h.**
